# A pathological role of the Hsp40 protein Ydj1/DnaJA1 in models of Alzheimer’s disease

**DOI:** 10.15698/cst2022.05.267

**Published:** 2022-05-09

**Authors:** Jelena Tadic, Julia Ring, Andrea Jerkovic, Selena Ristic, Marta Maglione, Jörn Dengjel, Stephan J. Sigrist, Tobias Eisenberg

**Affiliations:** 1 Institute of Molecular Biosciences, NAWI Graz, University of Graz, Graz, Austria.; 2 Field of Excellence BioHealth, University of Graz, Graz, Austria.; 3 NeuroCure Charité Berlin, Berlin, Germany.; 4 Institute for Biology, Freie Universität Berlin, Berlin, Germany.; 5 Department of Biology, University of Fribourg, Fribourg, Switzerland.; 6 BioTechMed-Graz, 8010 Graz, Austria.

**Keywords:** Alzheimer’s disease, amyloid beta 42, oligomers, heat shock proteins, HSP40, Ydj1, DnaJA1, yeast cell death, Drosophila, neurodegeneration

## Abstract

Alzheimer’s disease (AD) is the most common form of dementia with millions of people affected worldwide. Pathophysiological manifestations of AD include the extracellular accumulation of amyloid beta (Abeta) pep-tides, products of the proteolytic cleavage of the amy-loid precursor protein APP. Increasing evidence sug-gests that Abeta peptides also accumulate intracellular-ly, triggering neurotoxic events such as mitochondrial dysfunction. However, the molecular factors driving formation and toxicity of intracellular Abeta are poorly understood. In our recent study [EMBO Mol Med 2022 – e13952], we used different eukaryotic model systems to identify such factors. Based on a genetic screen in yeast and subsequent molecular analyses, we found that both the yeast chaperone Ydj1 and its human ortholog DnaJA1 physically interact with Abeta, facili-tate the aggregation of Abeta peptides into small oli-gomers and promote their translocation to mitochon-dria. Deletion or downregulation of this chaperone pro-tected from Abeta-mediated toxicity in yeast and Dro-sophila AD models, respectively. Most importantly, the identified chaperone is found to be dysregulated in post-mortem human samples of AD patients. Here, we aim to outline our key findings, highlighting pathological functions of a heat shock protein (Hsp) family member, which are generally considered protective rather than toxic during neurodegeneration. Our results thus chal-lenge the concept of developing generalized chaperone activation-based therapies and call for carefully consid-ering also maladaptive functions of specific heat shock proteins.

AD is considered as a "protein misfolding disor-der". Aggregation-prone Abeta accumulates in the brain of AD patients. Thus, the implication of Hsps in AD comes as no surprise. The cellular heat shock response is a highly conserved mechanism that acts as a critical defense in AD and other proteinopathies. Heat shock family proteins comprise chaperone function and assist the folding or unfolding of other proteins and peptides. Some of them bind misfolded or aggregation prone pro-teins, inhibiting early stages of aggregation or contrib-uting to the resolving of aggregates, respectively. There-fore, Hsps provide an attractive therapeutic target in AD. In addition to their function in proteostasis, Hsps are involved in translocation of their clients across different intracellular membranes, including mitochondria. Mito-chondria have a crucial role in generating ATP, amino-acid metabolism, calcium homeostasis, redox balance, lipid metabolism and programmed cell death. Disturb-ance of mitochondrial homeostasis can lead to several diseases, including neurodegenerative maladies. Cere-bral tissue has a high demand for energy supply and therefore critically depends on mitochondrial function, yet it is highly sensitive to oxidative stress, due to a low antioxidant capacity. The implication of dysfunctional mitochondria and oxidative stress in AD is well estab-lished. Accumulation of Abeta in mitochondria of AD patients, as well as Abeta driven mitochondrial damage have been reported several times. Yet, the pathological pathway of intracellular Abeta and regulation of its trans-location to mitochondria remain poorly understood.

To dissect the molecular mechanism of intracellular Abeta toxicity, we employed a humanized yeast model, expressing the Abeta peptide containing 42 amino acids (A42) C-terminally fused to enhanced green fluores-cence protein (EGFP-A42) accompanied by a linker re-gion in between to allow proper folding of EGFP. The EGFP tag provides the Abeta42 peptide with sufficient stability and we found that this construct induces typical hallmarks of cellular toxicity during AD. This includes oxidative stress, decreased ATP production, induction of mitochondria-dependent cell death, and finally accumu-lation of small-n soluble oligomers as well as high molec-ular weight Abeta species (i.e. high-n oligomers) partially located at mitochondria. Yeast provides an excellent eukaryotic model to study conserved pathways of cellu-lar stress and death, paired with powerful genetics. We validated our model using different peptide controls, i) empty vector control containing only EGFP, ii) Abeta containing 40 amino acids (A40), iii) a non-aggregating mutated form of Abeta42 (A42m2) and iv) the C-terminal peptide of Amyloid Precursor Protein 57 amino acids long, which all showed reduced or no toxicity compared to the Abeta42 peptide. With the intention to identify the crucial players modifying Abeta 42 cytotoxicity, we carried out two independent approaches: (i) we per-formed a genetic screen, testing the impact of knockout mutants of genes involved in cellular stress and cell death pathways and (ii) conducted a mitochondrial pro-teomic analysis of Abeta42 expressing yeast. Both ap-proaches revealed prominent involvement of mitochon-dria and mitochondrial function-associated proteins re-quired for Abeta42 toxicity. Moreover, a specific Hsp40 family member was identified to drive Abeta42 toxicity. Upon expression of EGFP-A42, this protein, namely Ydj1, was found to be upregulated. Deletion of *YDJ1* prevented cell death as assessed by measuring propidium iodide (PI) stained population. Us-ing a pull-down assay we showed that Ydj1 physically interacts with Abeta42, and subsequent analysis utilizing microscopy and immunoblotting demonstrated Ydj1’s impact on Abeta42 oligomerization and its translocation to mitochondria. These effects appear to be very specif-ic to this peptide, as deletion of *YDJ1* did not rescue for alpha-synuclein-induced cell death in yeast model for Parkinson´s disease nor did it show physical interaction to the mutated form of Abeta42 (A42m2). We observed similar findings utilizing an alter-native AD yeast model, targeting expressed Abeta42 to the ER/secretory pathway via Kar2 localization sequence. To further test if the human ortholog of Ydj1, namely DnaJA1, manifests the same Abeta42 related actions as Ydj1, we introduced DnaJA1-FLAG into the *YDJ1* deletion yeast strain co-expressing EGFP-A42. As a result, DnaJA1 restored Abeta42 in-duced toxicity in *ydj1* mutant yeast, re-storing Abeta42-induced cell death, translocation of EGFP-A42 to mitochondria and preventing EGFP-A42 degradation. In subsequent experiments, we aimed to corroborate our findings from yeast using alternative approaches. We performed in vitro assays, following the oligomerization properties of synthetic Abeta42 with and without presence of DnaJA1 protein. These assays revealed a strong implication of DnaJA1 in Abeta42 ag-gregation by accelerating low- and high-n oligomer for-mation detectable by immunoblotting, while its bacterial counterpart DnaJ delayed the formation of thioflavin-detectable larger aggregates and fibrils. To tackle the question of the human relevance of our findings, we evaluated DnaJA1 protein levels in human hippocampi of AD patients. We found differential DnaJA1 expression between demented and non-demented elderly controls, observing reduced DnaJA1 levels in AD brain tissue. We further extended our investigation to the genetically tractable model organism, Drosophila melanogaster. Pan-neuronal expression of Abeta42 to the secretory pathway led to upregulation of the Drosophila Ydj1 homologue, Droj2, similar to our yeast model system. Downregulation of Droj2 decreased neuronal Abeta42 accumulation, rescued shorter life span on manganese stress, and improved cognitive abilities as determined by aversive associative short term memory assay in a sex-specific manner. By assessing mitochondrial morphology changes upon reduction of Droj2 levels, we observed decreased solidity and circularity, thus being more simi-lar to the mitochondria of the wild type (Droj2 contain-ing) controls in Abeta42 expressing flies.

AD is one of the leading causes of mortality in devel-oped countries, thus becoming a significant social and health burden. The failure rate of potential AD drugs in clinical trials is enormous, and it appears at present that finding a cure for AD is a "mission impossi-ble". What could be the reason for this struggle? The moment AD is diagnosed, the brain tissue is already damaged to a remarkable extent, and further progres-sion of AD can only be delayed to some extent with available drugs. Consistently, a number of studies sug-gest that AD starts two or three decades before the first symptoms appear, making this dementia a “timing dis-ease” divided in different stages. It is a challenge to dis-sect the events happening in the brain tissue prior to the first symptoms. Therefore, model organisms provide us with a powerful tool to study the pre-symptomatic pro-cesses. Increasing evidence suggest that damaged and stressed mitochondria are among the early pathologies of AD. Apparently, this organelle plays a crucial role in the development of this disease. The Auwerx group re-cently demonstrated that the induction of mitochondrial proteostasis reduces Abeta aggregation in three differ-ent AD models, using a human neuroblastoma cell line, worms and transgenic mice. In parallel, another study reported that aggregation prone proteins are imported from the cytosol into mitochondria for their degradation. We therefore venture to hypothesize that Ydj/DnaJA1 may assist the cell to get rid of aggregation-prone Abeta, by translocating this peptide and its oligomers to mito-chondria for subsequent degradation (**Figure 1**). This would be consistent with previously re-ported cell-type specific vital functions of these Hsp40 proteins. However, if the balance of mitochondria-mediated proteostasis is disturbed or overwhelmed by high amounts of Amyloid proteins, this pathway could turn into a maladaptive response resulting in mitochon-drial dysfunction and cell death. Vice versa, mitochon-drial dysfunction itself, which occurs in aging and related co-morbidities, may drive such maladaptive response leading to a vicious cycle. In turn this would favor the progression to AD pathology, as observed in AD models examined in our study. Future experiments are needed to test this hypothesis and to demonstrate the potential dual role of Ydj1/DnaJA1 depending on the timing of disease progression. Thus, a critical question remains: When does the physiological function of this Hsp40 pro-tein turn pathological and what triggers this event in the human brain during AD development?

**Figure 1:**
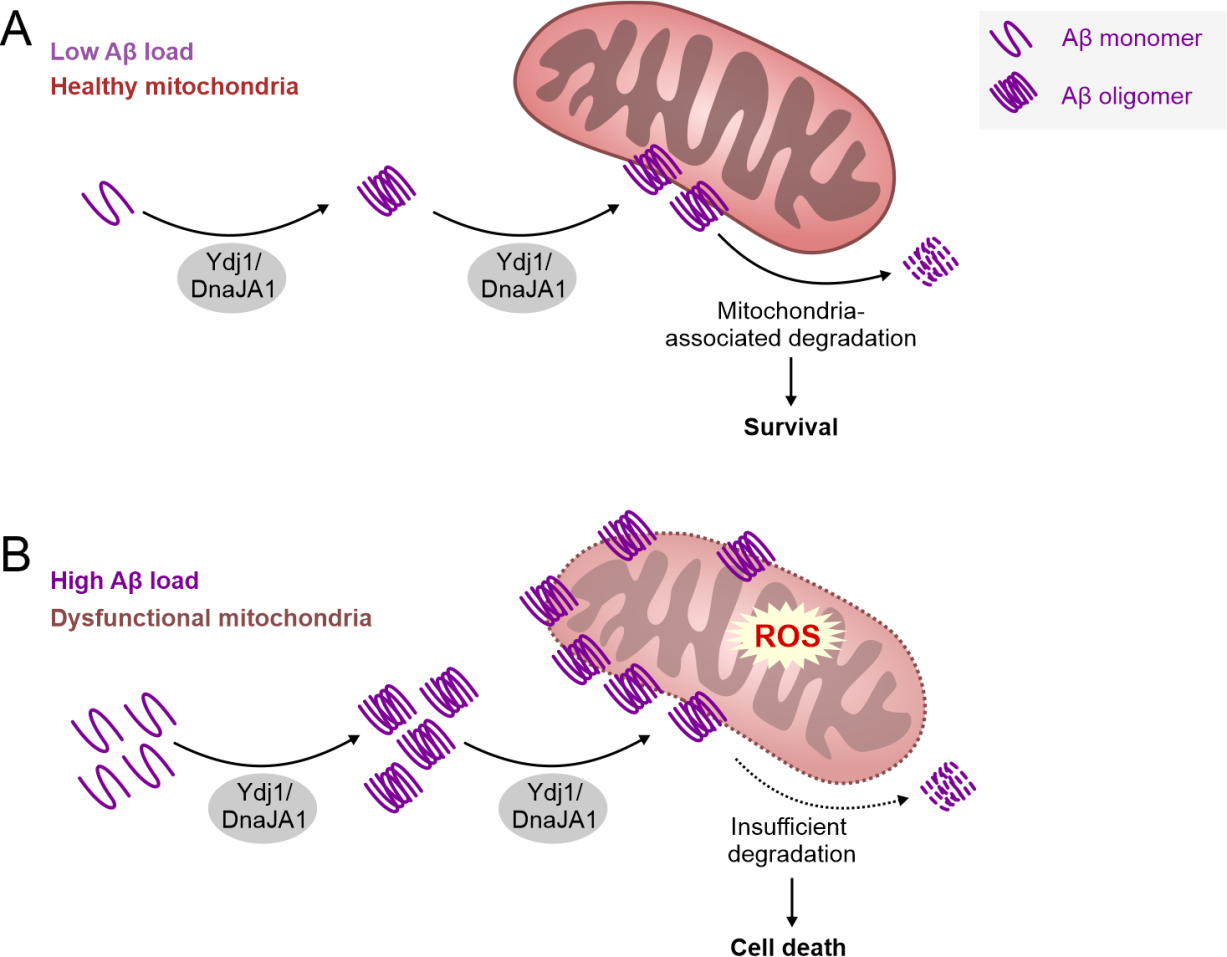
FIGURE 1: Hypothetical mechanism of Ydj1/DnaJA1’s role in Abeta toxicity. Abeta42 and its toxic oligomers are stabilized and translocated to mitochondria in a Ydj1/DnaJA1-dependent manner. We postulate that this behavior of Hsp40 family proteins is primed by the attempt to get rid of accumulating Abeta42 through mitochondria-dependent degradation (**A**). Nevertheless, in the case of high Abeta42 load or insufficient mitochondria-dependent degradation, Ydj1/DnaJA1 takes on a pathological role, leading to stressed mitochondria, eventually resulting in cell death promoting AD pathology (**B**).

In sum, using *in vitro* approaches combined with different *in vivo* models we identified a new key player and potential drug target of Abeta42-mediated toxicity. Ydj1/DnaJA1 counterintui-tively promotes the stabilization of toxic Abeta42 oligo-mers, rather than preventing the formation of toxic ag-gregates, and also regulates their transport to mitochon-dria. The dysregulation of human DnaJA1 in the brains of AD patients suggests that this Hsp may be involved in the etiology of AD, but future studies are needed to unravel the timing of these events in the human brain. Identify-ing the exact moment at which DnaJA1 is involved in the progress of AD could open new opportunities for the development of novel therapeutic or preventive strate-gies.

